# Transcultural Diabetes Nutrition Algorithm: Brazilian Application

**DOI:** 10.3390/nu7095342

**Published:** 2015-09-01

**Authors:** Fabio Moura, João Salles, Osama Hamdy, Walmir Coutinho, Deise Regina Baptista, Alexander Benchimol, Albert Marchetti, Refaat A. Hegazi, Jeffrey I. Mechanick

**Affiliations:** 1 Endocrinology and Metabolism Department, University of Pernambuco, Recife, Pernambuco 50100-130, Brazil; 2 Endocrine Unit, Department of Internal Medicine, Santa de Sao Paulo Medical School, Sao Paulo 04119-010, Brazil; E-Mail: jensalles@yahoo.com.br; 3 Joslin Diabetes Center, Harvard University, Boston, MA 02215, USA; E-Mail: osama.hamdy@joslin.harvard.edu; 4 Department of Endocrinology, Catholic University, Rio de Janeiro 22211-340, Brazil; E-Mail: wcoutinho@globo.com; 5 Department of Nutrition, Federal University of Parana, Curitiba, Parana 04566-905, Brazil; E-Mail: deiseregina@ufpr.br; 6 State Institute of Diabetes and Endocrinology, Rua Moncorvo Filho, 90, Centro, Rio de Janeiro 20211-340, Brazil; E-Mail: alexanderbenchimol@rjnet.com.br; 7 Department of Preventive Medicine and Community Health, Rutgers New Jersey Medical School, Newark, NJ 07101, USA; E-Mail: albertmarchetti@yahoo.com; 8 Research and Development, Abbott Nutrition, Columbus, OH 43219, USA; E-Mail: refaat.hegazi@abbott.com; 9 Division of Endocrinology, Diabetes and Bone Disease, Icahn School of Medicine at Mount Sinai, New York, NY 10029, USA; E-Mail: jeffreymechanick@gmail.com

**Keywords:** diabetes, prediabetes, obesity, nutrition therapy, physical exercise, algorithm, transcultural, Brazil, chronic non-communicable disease

## Abstract

The prevalence of obesity, pre-diabetes, and type 2 diabetes (T2D) is increasing worldwide, especially in the developing nations of South America. Brazil has experienced an exponential increase in the prevalence of these chronic non-communicable diseases. The rising prevalence is probably due to changing eating patterns, sedentary living, and a progressive aging of the population. These trends and their underlying causes carry untoward consequences for all Brazilians and the future of Brazilian public health and the healthcare system. Lifestyle changes that include healthy eating (nutrition therapy) and regular physical activity (structured exercise) represent efficient inexpensive measures to prevent and/or treat the aforementioned disorders and are recommended for all afflicted patients. Regrettably, the implementation of lifestyle changes is fraught with clinical and personal challenges in real life. The transcultural Diabetes Nutrition Algorithm (tDNA) is a therapeutic tool intended to foster implementation of lifestyle recommendations and to improve disease-related outcomes in common clinical settings. It is evidence-based and amenable to cultural adaptation. The Brazilian Diabetes Association, Society of Cardiology and Ministry of Health guidelines for nutrition therapy and physical exercise were considered for the Brazilian adaptation. The resultant tDNA-Brazil and its underlying recommendations are presented and explained.

## 1. Introduction

The prevalence of type 2 diabetes (T2D) has been increasing exponentially around the world, with 382 million patients in 2013, and a projection of 540 million by 2030 [1]. Likewise, prediabetes (the elevation of blood sugar levels, fasting or after the use of dextrose, (which still do not define T2D)), is rapidly on the rise worldwide [2]. These epidemiologic shifts have been caused by a growing prevalence of obesity, sedentary lifestyles, a high intake of refined sugar, and the consumption of fast foods or highly processed foods, all combined with an aging population [2].

In South America, the prevalence of T2D is rising faster than on any other continent, except for Asia [1]. In 2013, the estimated prevalence of T2D in South America was approximately 8% and that of prediabetes was about 7.4%. Type 2 diabetes was responsible for 11.5% of the deaths in the region, which corresponded to 226,000 people, and the total cost for the treatment of the disease was estimated to be US$26.2 billion, which corresponded to 13.5% of all resources spent on healthcare [1]. On the South American continent, Brazil has the largest population of patients with T2D, although the exact number is controversial. Regardless, based on observed trends, the prevalence of T2D in Brazil is expected to triple among individuals 45–64 years of age, the most affected segment of the population [2].

In the late 1980s, to better understand T2D and impaired glucose tolerance (IGT) in Brazil, an early multicenter study was performed in nine major Brazilian cities. Among residents 30–69 years of age, an estimated 7.8% had T2D and 7.5% had IGT [3]. The main factors associated with these high rates were obesity, aging, and family history. In 2003 in the city of Ribeirão Preto in the state of São Paulo, the prevalence of T2D was reported to be 12.2%; 7.7% for prediabetes [4]. In São Carlos, another city in the state of São Paulo, the prevalence was 13.5% for T2D and 5% for IGT [5]. Using official data from the Ministry of Health, Dias and Campos [6] reported a prevalence rate ≥10% for T2D in 24 of the 27 states in the Brazilian Federation during the period between 2002 and 2004, with an upward trend over time, reaching a general estimated prevalence rate of 11.7% in 2012.

Not only is the epidemiology of T2D in Brazil disconcerting, but control of the disease is similarly troubling, as glycemic control in Brazilian patients is reported to be far from currently recommended targets [7]. Only 26% of T2D patients had hemoglobin A1c (A1c) levels < 7%; average levels were found to be 8.6% ± 2.2%. Higher average levels were noted in the North and Northeast regions compared with South and Southeast areas. Lower levels of education, the use of insulin, disease duration, sedentary lifestyle, and non-Caucasian ethnicity were identified as risk factors for poor control [7].

Costs associated with the treatment of the disease were also very high [8]. The Brazilian Study on Diabetes Costs, the first of its kind in Brazil, estimated that the average annual per-patient cost was US$2108. Costs increased with the duration of disease, level of required care, and the presence of chronic diabetic complications. Patients with micro- or macro-vascular complications consumed US$3199 per year, whereas patients without any complications consumed US$1791 per annum. The largest component of costs (48%) was for medication [8].

Unfortunately, chronic complications are also high among patients with T2D in Brazil [9]. In a study of 927 outpatients, 33% had coronary ischemia and 36% had peripheral arteriopathy. Among those with microvascular complications, 37% had kidney disease (12% microalbuminuria) and 48% had peripheral retinopathy (15% proliferative retinopathy). Peripheral neuropathy was found in 36% of the patients.

Not surprisingly, in Brazil as in other areas around the world, there is an urgent need to improve the quality of prevention and treatment for needy patients. In this context, the Brazilian Diabetes Association (Sociedade Brasileira de Diabetes (SBD)) developed guidelines for T2D treatment [10], based on consensus recommendations from the American Diabetes Association (ADA), European Association for the Study of Diabetes (EASD) [11], and American Association of Clinical Endocrinologists (AACE) [12]. Across all of these recommendations, proper nutrition therapy (NT) and physical exertion are recognized as pillars of T2D treatment. Regrettably, in a “real-life” scenario, guidelines are difficult to implement, especially those related to behavior modifications for diet and exercise [13].

With the goal of enabling the application of nutrition therapy and regular physical activity in a simple, adequate, and effective manner, the transcultural Diabetes Nutrition Algorithm (tDNA) was created. The algorithm and related education initiatives resulted from collaborative work performed by an international group of healthcare experts with the aim of: (1) reinforcing the importance of nutritional interventions in the treatment of prediabetes and T2D; (2) encouraging healthy eating habits and regular physical exercise; and (3) adapting tDNA for regions of the world with culture specificities and preferences in mind. Delivering this intervention in a simple and practical format and maximizing physician and patient compliance with international recommendations are other key goals of the tDNA program.

The lead tDNA article describing developmental and transcultural processes that were used to create the original algorithm and its planned adaptations was published in 2012 [13]. Asian [14] and Indian [15] versions followed the lead paper, reporting the actual *transculturalization* of the original tDNA template to realities that exist in those two locations. Other adaptations and publications followed. See Table 1. Expressed in this current report is the Brazilian adaptation, which is based on the original template and the interactive collaboration of Brazilian experts in the field of obesity, diabetes, and other metabolic disorders in their home country.

**Table 1:** Transcultural Nutrition Algorithm Adaptations.

**Published**
Mechanick, J.; Marchetti, A.E.; Apovian, C.; Benchimol, A.K.; Bisschop, P.H.; Bolio-Galvis, A.; Hegazi, R.A.; Jenkins, D.; Mendoza, E.; Sanz, M.L.; *et al.* Diabetes-specific nutrition algorithm: A transcultural program to optimize diabetes and prediabetes care. *Curr. Diabetes Rep.* **2012**, *12*, 180–194. [13]
Su, H.-Y.; Huang, S.-Y.; Tsang, M.-W.; Mechanick, J.I.; Sheu, W.H.; Marchetti, A.; Task Force for Development of Transcultural Algorithms in Nutrition and Diabetes. Transculturalization of a diabetes-specific nutrition algorithm: Asian application. *Curr. Diabetes Rep.* **2012**, 12, 213–219. [14] Joshi, S.R.; Mohan, V.; Joshi, S.S.; Mechanick, J.I.; Marchetti, A. Transcultural diabetes nutrition therapy algorithm: The Asian Indian application. *Curr. Diabetes Rep.* **2012**, *12*, 204–212.[15] Hussein, Z.; Hamdy, O.; Chin Chia, Y.; Lin Lim, S.; Kumari Natkunam, S.; Hussain, H.; Yeong Tan, M.; Sulaiman, R.; Nisak, B.; Chee, W.S.; Marchetti, A.; Hegazi, R.A.; Mechanick, J.I. Transcultural Diabetes Nutrition Algorithm: A Malaysian Application. *Int. J. Endocrinol*. **2013**, *2013*.[16] Gougeon, R.; Sievenpiper, J.L.; Jenkins, D.; Yale, J-F.; Bell, R.; Després, J-P.; Ransom, T.P.P.; Camelon, K.; Dupre, J.; Kendall, C.; Hegazi, R.A.; Marchetti, A.; Hamdy, O.; Mechanick, J.I. The Transcultural Diabete Nutrition Algorithm: A Canadian Perspective. *Int. J. Endocrinol*. **2014**, *2014*. [17] Hamdy, O.; Marchetti, A.; Hegazi, R.A.; Mechanick, J.I. The Transcultural Diabetes Nutrition Algorithm Toolkit: Survey and Content Validation in the United States, Mexico, and Taiwan. *Diab. Technol. Therapeu*. **2014**, *16*, 378–384. [18] Nieto-Martínez, R.; Marante, D.; Hamdy, O.; Marulanda, M.I.; Marchetti, A.; Hegazi, R.A.; Mechanick, J.I. Transcultural Diabetes Nutrition Algorithm (tDNA): Venezuelan Application. *Nutrients* **2014**, *6*, 1333–1363. [19] Galvis, A.B.; Hamdy, O.; Pulido, M.E.; Haje, V.A.R.; Molina, H.A.L.; Martínez Sánchez, M.E.; González Bárcena, D.; y de Yta, T.H.; Marchetti, A.; Hegazi, R.A.; Mechanick, J.I. Transcultural Diabetes Nutrition Algorithm: The Mexican Application. *J. Diabetes Metab.* 5, 423 doi:10.4172/2155-6156.1000423 [20]
**In Development**
Cecilia A. Jimeno, Roberto C. Mirasol, Osama Hamdy, Albert Marchetti, Refaat A.Hegazi, Jeffrey I. Mechanick. Transcultural Diabetes Nutrition Algorithm (tDNA): The Philippine Application.Panamanian, Costa Rican, Colombian, and Middle Eastern Applications
Panamanian, Costa Rican, Colombian, and Middle Eastern Applications

## 2. Experimental Section—Methodology

To undertake the Brazilian adaptation, nationally recognized healthcare professionals with expertise in endocrinology, physical medicine, and nutrition were identified from various regions of the country. Each expert was contacted, briefed on the project, and questioned about his or her interest in participating in the tDNA initiative. Based on responses, invitations were extended to a select group of these specialists, requesting their involvement. A task force was formed when a group (*n* = 5) sufficient for advisory activities was established.

**Figure 1:**
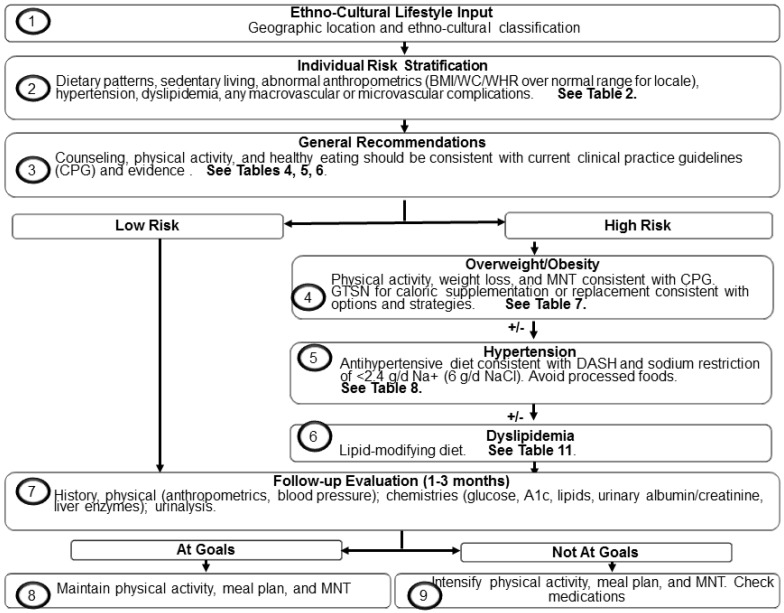
Transcuture Diabetes Nutrition Algorithm (tDNA)—Brazilian Application.

At meetings and through subsequent interactions, members of the task force reviewed the original tDNA template and provided culturally meaningful data and opinion to adapt the algorithm to the Brazilian condition. Members discussed the influence of various risk factors and comorbidities (e.g., obesity, hypertension, dyslipidemia, prediabetes and cardiovascular disease) in the assignment of patients to specific algorithmic pathways. Similarly, task force members deliberated over the merits, practicality, and inclusion of specific measures (e.g., body weight *vs.* waist-to-hip ratio (WHR)), tests (e.g., fasting blood glucose *vs.* (A1c)), and nutritional therapies (e.g., calorie supplementation or substitution with prepared diabetes-specific formulas) that would be cited in their final recommendations. Transcultural factors influencing dietary practices, food availability and selection, and T2D healthcare interventions were also considered. Thereafter, attending task force members and a tDNA executive committee reviewed summaries of the proceedings in order to finalize the algorithm (Figure 1) and this report. The remainder of this paper represents an amalgam of their deliberations, conclusions, and recommendations and presents for the first time the tDNA-Brazilian Application. 

## 3. Transcultural Findings

### 3.1. Eating Patterns in Brazil

As in other cultures, eating habits in Brazil have been changing through the years [21]. Between 1988 and 1996, a progressive increase was observed in the consumption of saturated fat, sugar, and soft drinks, while the consumption of complex carbohydrates, fruits, and vegetables declined in metropolitan regions [22]. Beef and dairy products were the main sources of saturated fat. Sweets and desserts were the main sources of sugar [23].

According to data from Vigitel 2012 [24], only 22.7% of adults eat at least five daily servings of fruits and greens, while 31.5% consume milk and whole dairy products and 26.5% consume soft drinks daily. An important contributing factor is the increase in the habit of dining outside of the home. Forty-three percent of Brazilians report eating out at least once a week [25], which contributes to a high intake of calories, saturated fat, *trans* fat, and simple sugars [25]. The most consumed foods were alcoholic beverages, fried foods, pizzas, soft drinks, sandwiches, sweets, and desserts. These observations were most strongly associated with young adults from lower socio-economic groups.

Another remarkable eating habit of Brazilians is frequent snacking [26]. Seventy-four percent of the Brazilian population snacks at least once a day, especially in the late afternoon. Twenty-three percent of people are considered “heavy snackers” (having three or more snacks a day), and the foods most often consumed are coffee with sugar, desserts, sweets, fruits, and savory pastries. A direct association between the frequency of snacks and increased calorie intake was also reported. The average caloric intake was 2334 kilocalories among snackers compared to 1546 kilocalories in non-snackers. Moreover, in larger cities, workers start early in the morning, frequently missing breakfast and snacking instead. For these individuals, evening meals tend to be large, as family members are together and have more time to relax.

### 3.2. Sedentary Lifestyles in Brazil

Sedentary lifestyles are also a major problem in Brazil. According to the Brazilian Diabetes Association, only 33.5% of Brazilian adults reach the recommended 150 min a week of light to moderate physical activity or at least 75 min of vigorous physical activity, which was higher for men (41.5%) than for women (26.5%) [27,28]. Age and lower education level were identified as risk factors for sedentary living. Although data about physical activity among persons with T2D are scant, they suggest a higher prevalence of sedentary lifestyles compared with those without T2D. In a study sample evaluated by Cunha *et al.* [29], 82% of adults and 89% of seniors (over age 65) with T2D reported sedentary lifestyles. In a study by Duarte *et al.* [30], 30.7% of patients were sedentary (no physical activity), 60.6% were considered moderately active, and only 8.7% were highly active (physical activity performed at least five times a week).

### 3.3. Prevalence of Overweight and Obesity in Brazil

Between 1975 and 2003, the prevalence of obesity increased by 92% for men and 63% for women [31]. In a survey performed by the Ministry of Health in 2009, the frequency of overweight persons was 46.6% and obesity was 12% in the general population [32]. In 2012, those numbers increased to 51% and 17%, respectively [24]. Age, gender, and education level correlated with risk [24,32,33,34]. For example, among men, obesity prevalence was almost three times higher for those 55–64 years of age compared to those aged 18–24 years (22% *vs.* 8.1%, respectively), with lower levels for the 65 years or older population (17.4%). Among women, the frequency of obesity was four times higher for 55–64 year old individuals compared to those aged 18–24 years (25.1 and 6.9%, respectively), with a decline in frequency noted for those older than 65 years. An inverse association was reported between overweight and education, especially among women: 50.0% in the lower education group compared to 31.1% in the higher education group [24].

### 3.4. Overweight and Obesity among Brazilians with T2D

In a Brazilian multicenter study of 2519 patients with T2D, the average body mass index (BMI) of study subjects was 28.3 ± 5.2 kg/m^2^ [33], which was slightly lower than that reported in studies from the United Kingdom (29.9 kg/m^2^) and the United States (32.3 kg/m^2^), but higher than those noted in Asia [14] and India [15]. When stratified by BMI, 1.2% of Brazilians with T2D had low body weight (BMI < 18.5 kg/m^2^), 23.8% had normal weight (BMI 18.5%–24.9%), 42.1% were overweight (BMI 25–29.9 kg/m^2^), and 32.9% were obese (BMI ≥ 30%); that is, 75% of assessed patients were overweight/obese [33]. No difference was observed in the prevalence of low body weight among the different regions of the country (*p* = 0.35) [33]. There was, however, a higher prevalence of obesity in the Southeast and South regions, compared to the Northeast region (*p* < 0.001) [33]. Patients in the Northeastern region had an average BMI of 26.4 ± 4.7 kg/m^2^ *vs.* 27.9 ± 4.8 kg/m^2^ in the Mid-west, 29.2 ± 5.1 kg/m^2^ in the Southeast, and 29.4 ± 5.4 kg/m^2^ in the South.

### 3.5. Implications of Increased Longevity on Sarcopenia and T2D

The increase in longevity that has occurred during the past several decades in most developed countries is associated with several health implications [34]. Sarcopenia, defined as decrease of lean body mass and muscular strength that is associated with aging, is one consequence [35]. Although the precise prevalence of sarcopenia can be debated, it could affect as many as 64% of men over 65 years of age in the United States [36].

Likewise, the increase in T2D prevalence is partly a consequence of the secular trend in life expectancy [37]. Currently, the prevalence of T2D in the population aged ≥ 65 years varies from 23% in South Korea to 33% in the US [38]. By 2030, the expected number of seniors with T2D will be four times higher in those countries [38]. Patients with T2D, especially those with longer duration disease and worse glycemic control, have sarcopenia more often, more severely, and earlier than the general population [39].

Brazil is going through broad and fast demographic transitions, with an exponential increase in the senior population [40]. By 2050, the estimated number of people aged 60 years or older is projected to be greater than the estimated number of people 30 years of age or younger. Sarcopenia is expected to become a major public health issue, demanding additional costs for the healthcare system. At present, no prevalence data for sarcopenia could be found in Brazil.

### 3.6. Hypertension

Hypertension (blood pressure > 140/90 mmHg) is one of the most important modifiable risk factors for cardiovascular disease (CVD) [41]. Like other chronic non-communicable diseases its prevalence is increasing in Brazil. According to the Brazilian Cardiology Society, the prevalence rate of hypertension among Brazilian adults (age between 18–60 years old) is about 32.5%, increasing to more than 50% among the elderly (older than 60 years) [41]. Obesity, especially central obesity, sedentary lifestyle, and a diet rich in salt and saturated fats are risk factors for hypertension [42]. Weight loss, regular physical activity, and an adequate diet [43,44] with low sodium and alcohol intake, together with a high intake of potassium, calcium, magnesium, fiber, and monounsaturated fats (MUFA) (e.g., Dietary Approaches to Stop Hypertension (DASH) diet) can help in prevention and treatment [43,44].

### 3.7. Dyslipidemia

Dyslipidemia is another important modifiable risk factor for CVD [45,46]. Although scant data are available to pinpoint its precise prevalence in Brazil, one study analyzed 49,385 patients living in the eight largest cities of the country and found that 16.9% of adults had the disorder [47]. Better economic status and higher BMI are identified risk factors [46]. The Brazilian cardiology society recommends nutrition therapy, increased physical activity, and smoking cessation for all patients with dyslipidemia [41].

### 3.8. Nutrition Therapy

The concept of nutrition therapy (NT) involves dietary modification as an important therapeutic tool for the prevention and treatment of chronic non-communicable disease [13], especially T2D. It implies individualized nutritional assessment and follow-up, adapted to the required amount (calories) and quality (composition) of food. The efficacy of nutritional interventions for primary prevention of T2D among patients with prediabetes and high risk of progression has been demonstrated in a wide variety of populations. In the Diabetes Prevention Program (DPP) study [48], lifestyle changes reduced the incidence of T2D by 58% and were more effective than metformin in preventing the disease. In the Finnish Diabetes Prevention Study [49], patients in the intervention group lost more weight (3–4.5 kg) than patients in the control group and experienced a 55% decrease in the incidence of T2D. In the Da Qing study [50], dietary intervention as primary prevention decreased the risk of developing T2D by 31%.

Regarding secondary prevention, slowing the progression of disease in patients with established T2D, Esposito *et al.* [51] demonstrated that an adequate nutritional intervention fostered better glycemic control and less need for oral hypoglycemic agents. Kulkarni *et al.* [52] has shown an average absolute decrease of 0.8% in A1c in patients with T2D who were being treated with oral hypoglycemic agents. A Brazilian study has shown decreased body weight and improved glycemic control, defined as weekly average blood sugar less than 150 mg/dL, in 75% of patients with T2D who were prescribed intensive nutritional intervention (decrease in Calories, increase in fiber, and monosaturated fat) when compared to a “standard eating” group in which only 30.7% of patients had the same results [53]. The ADA recognizes the essential role of nutrition therapy for diabetes management: (1) to promote and support healthful eating patterns, emphasizing a variety of nutrient-dense foods in appropriate portion sizes; (2) to improve overall health; and (3) to attain individualized glycemic, blood pressure, and lipid goals by achieving and maintaining body weight goals in order to delay or prevent the complications of diabetes [54].

### 3.9. Glycemia Targeted Specialized Nutrition (GTSN) for Metabolic Disorders

Glycemia targeted specialized nutrition products are meal replacements that are fortified with vitamins and minerals intended to deliver a well-defined number of calories [55]. They are consumed daily to replace snacks, one or more meals and are designed to help individuals maintain healthy diets. Liquid or solid (bars) products used in conjunction with conventional diets are a partial meal replacement [55,56], which were first prescribed for weight reduction and proven to be effective. In a meta-analysis of six studies, weight loss associated with partial meal replacement was shown to be superior to that achieved with conventional diets [56].

Glycemia targeted specialized nutrition represents an evolution in the concept of meal replacement. It generally consists of a scientifically designed formula that is composed of macro- and micro-nutrients adequate for the needs of patients with metabolic abnormalities (hyperglycemia, insulin resistance, and atherogenic lipid profile) [55,56,57,58,59,60]. It combines low glycemic index carbohydrates with fiber, MUFA, easily-absorbed proteins with high biological value, vitamins, and microminerals in controlled-calorie portions. The composition is versatile enough to be used as a replacement meal for patients on a hypocaloric diet for weight loss or as a dietary supplement for patients in need of a hypercaloric diet to increase body mass [55].

In the Look Ahead study [58], which included more than 5000 patients with T2D and obesity who were randomized to an intensive lifestyle intervention or to a control (diabetes support and education), one of the main factors associated with weight loss and metabolic improvement was the total number of meals replaced. Moreover, patients with metabolic syndrome using meal replacement therapy with protein rich GTSN lost more body fat and less lean mass than patients consuming a conventional diet [54].

In a study conducted by Tatti *et al.* [60] among elderly patients who had poorly-controlled T2D with sarcopenia, the use of GTSN as meal supplementation in a hypercaloric diet combined with increased physical exertion resulted in better glycemic control and increased muscle mass. In the Shanghai study [61] involving slightly overweight or normal weight patients, improvement in glycemic control was associated with modest weight loss and marked decrease in waist circumference.

### 3.10. Role of Physical Activity in Pre-T2D and T2D

Physical activity is another fundamental component of lifestyle modification. Structured programs have been proven to be efficient in preventing the progression of prediabetes to T2D and also for the treatment of established T2D [62]. In the Da Qing study, patients in the physical exercise group reduced their risk of developing T2D by 46% [50,63]. Hu G. *et al.* [64] showed an inverse relationship between the practice of regular physical exercise and the risk of developing T2D in patients with or without excessive weight. Moreover, in patients with established T2D, regular physical exercise improved glycemic control; Boule *et al.* [65] have shown an absolute 0.64% decrease in A1c levels among patients with T2D after implementing a structured exercise plan. All-cause mortality also appeared lower in patients with T2D who exercised regularly [66,67]. Although most of the studies for prevention and treatment of T2D involved aerobic exercises, resistance exercises are also important [68]. Moreover, a combination of aerobic and resistance exercises seems to be the most effective, considering that aerobic exercises improve insulin resistance and decrease body fat, while resistance exercises increase muscle mass, revert sarcopenia, improve balance, and decrease the risk of falling, which is especially important among elderly patients with T2D [69]. In the Why Waitstudy [69], the combination of aerobic and resistance exercises, along with longer-duration weekly activities goals, resulted in substantial and sustained weight loss. In Brazil, Vancea *et al.* [70] have shown that patients with T2D who were prescribed an exercise program consisting of 30 min of walking, five times a week, at 70% maximum heart rate decreased body fat, waist circumference and fasting blood glucose at the 20 week intervention point. The Brazilian Society of Diabetes (Sociedade Brasileira de Diabetes—SBD) recommends moderate daily physical exercise five times a week or 150 min (30 min per day) or intense physical exercise 75 min per week (25 min three times per week). It also recommends screening for silent CVD and diabetes complications [70].

## 4. Results: tDNA—Brazilian Application

Through a developmental process of more than three years, the original tDNA template was conceived, refined, and contextually validated, then adapted to various cultures on a global scale. International adaptations involved scores of meetings and reviews with a revolving and expanding faculty, which has led to numerous related publications in several peer-review journals. See Table 1. For detailed descriptions of the overall methodology and the evidence and recommendations that supported it, which are too extensive to report here, please refer to the lead publication and its companion pieces [13,14,15]. In Brazil, a national team of experts in nutrition, physical medicine, and endocrinology employed the same principles and methods to adapt the tDNA template to circumstances in that country and formed their recommendations accordingly. Those recommendations are found in the following section. See the Figure: Transcultural Diabetes Nutrition Algorithm (tDNA)—Brazilian Application.

### 4.1 Recommendations Within tDNA—Brazilian Application

#### Recommendation 1

Eating patterns and diet composition influence the risks of developing chronic non-communicable diseases such as obesity, pre-diabetes, and T2D, and always should be thoughtfully considered when assessing patients.

#### Recommendation 2

All patients should be evaluated with a full clinical history and a complete physical examination focused on nutritional status, fat distribution, and co-morbidities. BMI, waist circumference (WC) and blood pressure should always be recorded. Fasting plasma glucose and A1c testing for prediabetes or T2D should always be performed, a lipid panel also. At the end of the consultation, a risk assessment should be conducted (Table 2, Table 3 and Table 4). 

**Table 2:** BMI, WC, and Diabetes Risk for Brazilians Patients.

	BMI (kg/m^2^)	Level of Obesity	Disease Risk	
			WC Male ≤ 102 cm Female ≤ 88 cm	WC Male ≤ 102 cm Female ≤ 88 cm
**Underweight**	<18.5			
**Normal**	18.5–24.9			
**Overweight**	25.0–29.9		Elevated	High
**Obesity**	30.0–34.9	I	High	Very high
	35.0–39.9	II	Very high	Very high
**Extreme Obesity**	≥40	III	Extremely high	Extremely high

BMI, body mass index; WC, waist circumference, waist measured at the level of the anterior superior iliac crest.

**Table 3:** A1c Assessment for the Diagnosis of Prediabetes and T2D.

Diagnosis/Condition	A1c	Risk
**Without Diabetes**	4%–5.6%	-
**Prediabetes**	5.7%–6.4%	High
**Diabetes**	≥6.5%	Very High
**Controlled Diabetes**	<7%	Very High
**Uncontrolled Diabetes**	≥7%	Extremely High

A1c, hemoglobin A1c; (1) The Brazilian Society of Diabetes recommends that A1c alone should not be used for T2D and Pre Diabetes diagnosis; (2) High performance liquid chromatography is the best assay for A1c measure.

**Table 4:** Screening Before Beginning a Structured Physical Activity Program.

Condition	Subjects	Comments
**Silent Cardiovascular Disease**	All patients with CND older than 35 years old	Clinical history (exercise dyspnea, chest discomfort), physical examination (blood pressure, cardiac rhythm, arterial pulses), ECG, Echocardiogram, cardiac stress total
**Autonomic Neuropathy**	Diabetic patients	Clinical history, physical examination (postural hypotension, tachycardia)
**Peripheral Neuropathy**	Diabetic patients	Clinical history (paresthesia, allodynia), physical examination (hypoesthesia)
**Retinal**	Diabetic patients	Ophthalmologic evaluation (dilated fundoscopy), retinal angiography
**Nephropathy**	Diabetic patients	Albumin/creatinine ratio

ECG, electrocardiogram; CND, chronic non-communicable disease.

#### Recommendation 3

Nutrition therapy is crucial in the prevention and treatment of T2D and other non-communicable chronic diseases and should be recommended for all patients, always respecting their social, cultural and economic circumstances.

#### Recommendation 4

Nutrition therapy, with regular physical activity, should be customized to meet the unique clinical needs and conditions of individual patients, considering BMI, age, gender, glycemia, and the presence of co-morbidities, such as hypertension and dyslipidemia, and disabilities (Table 5, Table 6, Table 7, Table 8, Table 9, Table 10 and Table 11). 

**Table 5:** Physical Activity Recommendations.

Type of Activity	Examples	Frequency	Duration
**Moderate Aerobic**	Walking Swimming Dancing Cycling	≥5 days/week	≥30 min
**Intense Aerobic**	Walking Swimming Dancing Cycling	≥3 days/week	≥25 min
**Resistance**	Resistance bands, Hand weights, Weight training equipment	≥2 day/week	≥10 min
**Stretching**	Calf stretching Pilates Yoga	After each activity session	≥5–10 min

**Table 6:** Nutritional Guidelines for T2D by Brazilian Diabetes Association.

	Recommendations (Daily)
Carbohydrates	45%–60% of caloric intake
Sucrose	Less than 10% of caloric intake
Fructose	No addiction
Fibers	20 g (minimal) 14 g/1000 Kcal (ideal)
Fats	Less than 30% of caloric intake
Saturated fats	Less than 7% of caloric intake
Trans fat	Less than 2 g (maximal) No *trans* fat (ideal)
Polyunsaturated fats	10% of caloric intake
Monounsaturated fats	Individualized. Increase the ingestion
Cholesterol	Less than 200 mg
Proteins	15%–20% of caloric intake
Vitamins and minerals	No specific recommendations for this population
Sodium	Less than 2.400 mg

**Table 7:** Caloric Needs and GTSN Recommendations Based on Gender, BMI and A1c.

Nutritional State (BMI)	A1c	Gender	Meal Plan	Specialized Nutrition in Blood Sugar Control
Overweight/Obesity BMI 25–29.9	Any	Female	Plan 1 1200 Calories	≤3
		Male	Plan 2 1500 Calories	≤3
Overweight/Obesity BMI >30	Any	Female	Plan 2 1500 Calories	≤3
		Male	Plan 3 1800 Calories	≤3
Normal Weight BMI 18.5–24.9	<7%	Female	Plan 3 1800 Calories	Clinical criteria
		Male	Plan 4 2200 Calories	Clinical criteria
	≥7%	Female	Plan 3 1800 Calories	≤2
		Male	Plan 4 2200 Calories	≤2
Underweight BMI <18.5	Any	Male/Female	Plan 5 2200 Calories	≤3

GTSN, glycemia targeted specialized nutrition; BMI, body mass index; A1c, hemoglobin A1c.

**Table 8:** Classification of Hypertension by Brazilian Cardiology Society.

Classification of Blood Pressure	Systolic Blood Pressure	Diastolic Blood Pressure
Excellent	<120 mmHg	<80 mmHg
Normal	<130 mmHg	<85 mmHg
Pre-hypertension	130–139 mmHg	85–89 mmHg
Hypertension Stage 1	140–159 mmHg	99–99 mmHg
Hypertension Stage 2	160–179 mmHg	100–109 mmHg
Hypertension Stage 3	180 mmHg	110 mmHg
Systolic Hypertension	>140 mmHg	<90 mmHg

**Table 9:** Brazilian Cardiology Society Nutritional Recommendations for Hypertension.

Choose foods that have minimal saturated fat, cholesterol, and total fat, for example, lean meat, poultry, and fish, using them in moderation.
Eat varied fruits and vegetables, approximately eight to ten servings per day (one serving is equal to an average shell).
Include two or three servings of nonfat or semi-skimmed dairy per day.
Prefer whole foods such as bread, whole cereals and whole grains, or whole wheat pasta.
Consume oil (olive oil, nuts), seeds, and grains, four to five servings per week (one serving is equal to 1/3 cup or 40 g of nuts, two tablespoons or 14 grams of seeds or 1/2 cup of beans or cooked and dried peas)
Reduce added fats. Use light margarine and unsaturated vegetable oils (such as olive, soy, corn, canola oil).
Avoid adding salt to food. Also avoid ready-made sauces, broths, and industrial products.
Reduce or avoid consumption of sweets and sugary drinks.

**Table 10:** Classification of Cholesterol and Triglycerides by Brazilian Cardiology Society.

Blood lipids	Range (mg/dL)	Classification
**Total C**	<200 mg/dL	Excellent
	201–239 mg/dL	Borderline
	>240 mg/dL	High risk
**LDL-C**	<100 mg/dL	Excellent
	101–129 mg/dL	Normal
	130–159 mg/dL	Borderline
	160–189 mg/dL	High risk
	>190 mg/dL	Very high risk
**HDL-C**	>60 mg/dL	Excellent
	<40 mg/dL	Low
**TG**	<150 mg/dL	Excellent
	151–200 mg/dL	Borderline
	201–499 mg/dL	High
	>500 mg/dL	Very high
**Non HDL**	<130 mg/dL	Excellent
	131–159 mg/dL	Borderline
	160–189 mg/dL	High
	>190 mg/dL	Very high

C, cholesterol; LDL-C, low density lipoprotein cholesterol; HDL-C, high density lipoprotein cholesterol; TG, triglyceride; (1) Appropriate lipids levels must be individualized for each patient according to cardiovascular CVD risk; (2) CVD risk assessment must take account the sum of all risk factors (T2D, hypertension, dyslipidemia, smoking), using a CVD risk score, for example, the Framingham score.

**Table 11:** Brazilian Cardiology Society Nutritional Recommendations for Dyslipidemia.

Recommended Consumption			
Foods	Daily	Moderate	Occasional
Cereals	Whole grains	White bread, crackers, rice, pasta, sugary cereals.	Sweet breads, cakes, pies
Vegetables	Raw and cooked vegetables	--------------------------	Buttered vegetables
Fruits	Fresh fruits	Dried fruits, jellies	-------------------------
Sweets and Sweeteners	Non caloric	Honey, chocolate	Cakes, pies
Meat, fish, poultry	Fish, chicken without skin	Lean meat, seafood	Sausages, salami, canned meat, viscera.
Milk, Eggs	Nonfat (skimmed) milk and yogurt, cooked egg white	Low fat (semi- skimmed) milk, white cheese.	Yellow and cream cheese, egg yolk, milk, whole yogurt
Sauces	Vinegar, mustard, olive oil	--------------------------	Butter, solid margarine, pig and trans fat, coconut oil.
Nuts, Seeds	Small amounts: less than 30 g per day	All	Coconut
Food Preparation	Grilled, roasted, or steam	Baked	Fried

#### Recommendation 5

In real life, lifestyle modifications such as changes in dietary habits and physical activities are difficult to implement. To increase the likelihood of success, local habits and preferences should be considered. Food selection should be based on cultural factors, in observance of general recommendations from the American Diabetes Association (ADA), EASD, and AACE.

#### Recommendation 6

Determine Caloric Needs: Based on the patient’s feeding habits and current weight, determine caloric needs for a safe and sustainable weight loss (1200, 1500, 1800 calories) for individuals with overweight or obesity. The goal should be a weight loss of 0.5–1 kg per week. For underweight or normal weight patients, a higher caloric intake should be advised. (1800 to 2200 calories).

Select A Healthy Food Plan: Based on ADA and AACE recommendations for nutrition therapy, the patient could be referred to a registered dietitian, if available, for nutritional guidance and continuous support. See general food recommendations in Table 6.

#### Recommendation 7

The use of a “healthy, high-quality diet” composed of complex carbohydrates and rich in fiber (whole grains, whole bread roots, fruits, and vegetables), with a very low amount of sugar (soft drinks and desserts), low in unsaturated and *trans* fats, rich in monounsaturated and polyunsaturated fats (olive oil, low fat dairy products, and nuts) and with an adequate amount of protein from good sources (low fat meat, poultry, and fish) should be endorsed for all patients. The ingestion of salt should not exceed more than 2.4 g per day. 

#### Recommendation 8

Glycemia targeted specialized nutrition (GTNS) can be helpful in patients with metabolic disturbances. It provides a safe and practical option to improve diet quality with a set caloric goal. It can be used as partial meal replacement for caloric restriction and metabolic control in patients who are overweight/obesity or for caloric supplementation and metabolic control in patients who are underweight or experience sarcopenia. Patients with normal weight and elevated glucose levels can use it for metabolic control. 

#### Recommendation 9

The use of GTSN should be considered according to the nutritional status of the patient. In that context, based on the results of the Look Ahead and Why Wait studies, as well as the eating habits of Brazilians, a group of Brazilian specialists recommended up to three replacements per day, along with two main meals (breakfast, lunch or dinner) and a snack, targeting a 5%–10% weight loss and metabolic improvement. In light of frequent behaviors reported by Brazilian patients (*i.e.*, late afternoon snacking and consuming large amounts of highly caloric foods), the group suggests that the use of GTSN in the late afternoon might be considered. For patients with normal weight who are poorly controlled, the recommendation is two replacements per day; one as a main meal and one as a snack. For patients with normal weight who are well controlled, the use of GTNS is optional. In those who are underweight, possibly with sarcopenia, and in need of a hypercaloric diet, the recommendation is using GTNS as a nutritional supplement for snacks or as an addition to main meals.

#### Recommendation 10

Regular physical activity is very important in the prevention and treatment of obesity, pre-diabetes and T2D. A structured physical exercise program with 120–150 min of aerobic activity per week combined with resistance exercises, at least twice a week, should be recommended for all patients. Resistance exercises are especially beneficial for elderly patients to improve balance, prevent or minimize falls, and abate sarcopenia.

#### Recommendation 11

Before initiating a structured physical exercise program, all patients should be screened for asymptomatic CVD. Special care must be taken with those with long standing T2D who also should be screened for diabetic complications (proliferative retinopathy, severe peripheral neuropathy, and autonomic neuropathy) in order to prevent further damage.

#### Recommendation 12

Complementary lifestyle changes, such as smoking cessation, decreased alcohol intake to less than 140 g of alcohol for women and 280 g for men, and behavioral modifications to address sleep quality and mood disorders, should be recommended for all patients.

## 5. Conclusions

Adapting the original tDNA template to circumstances in Brazil and other international locations led to the recognition that local primary care practitioners along with other local healthcare professionals should actively promote healthy lifestyles to their patients. Professional involvement should include: (1) simple, quick, and effective methods to assess risky nutritional and physical activity behaviors and (2) related strategies to help change those behaviors in a sustainable way. Foods for Brazilians with T2D should be nutritionally adequate, culturally acceptable, and economically accessible in order to make healthy choices a daily occurrence and also to facilitate the adoption of eating behaviors that enhance diabetes care. Additionally, simple tools should be available to help healthcare professionals evaluate the physical activity habits of their patients and then support viable solutions that enable patients to increase their daily activity and to elevate their regular exercise level. The Brazilian tDNA is a step in this direction.
